# The role of mitochondrial reactive oxygen species, NO and H_2_S in ischaemia/reperfusion injury and cardioprotection

**DOI:** 10.1111/jcmm.15279

**Published:** 2020-05-08

**Authors:** Ioanna Andreadou, Rainer Schulz, Andreas Papapetropoulos, Belma Turan, Kirsti Ytrehus, Peter Ferdinandy, Andreas Daiber, Fabio Di Lisa

**Affiliations:** ^1^ Laboratory of Pharmacology Faculty of Pharmacy National and Kapodistrian University of Athens Athens Greece; ^2^ Institute for Physiology Justus‐Liebig University Giessen Giessen Germany; ^3^ Department of Biophysics Faculty of Medicine Ankara University Ankara Turkey; ^4^ Department of Medical Biology UiT The Arctic University of Norway Tromso Norway; ^5^ Department of Pharmacology and Pharmacotherapy Semmelweis University Budapest Hungary; ^6^ Pharmahungary Group Szeged Hungary; ^7^ Molecular Cardiology Center for Cardiology 1 University Medical Center of the Johannes Gutenberg University Mainz Germany; ^8^ Department of Biomedical Sciences Università degli Studi di Padova Padova Italy

**Keywords:** cardioprotection, heart, hydrogen sulphide, ischaemia, mitochondria, nitric oxide, reactive oxygen species, reperfusion

## Abstract

Redox signalling in mitochondria plays an important role in myocardial ischaemia/reperfusion (I/R) injury and in cardioprotection. Reactive oxygen and nitrogen species (ROS/RNS) modify cellular structures and functions by means of covalent changes in proteins including among others *S*‐nitros(yl)ation by nitric oxide (NO) and its derivatives, and *S*‐sulphydration by hydrogen sulphide (H_2_S). Many enzymes are involved in the mitochondrial formation and handling of ROS, NO and H_2_S under physiological and pathological conditions. In particular, the balance between formation and removal of reactive species is impaired during I/R favouring their accumulation. Therefore, various interventions aimed at decreasing mitochondrial ROS accumulation have been developed and have shown cardioprotective effects in experimental settings. However, ROS, NO and H_2_S play also a role in endogenous cardioprotection, as in the case of ischaemic pre‐conditioning, so that preventing their increase might hamper self‐defence mechanisms. The aim of the present review was to provide a critical analysis of formation and role of reactive species, NO and H_2_S in mitochondria, with a special emphasis on mechanisms of injury and protection that determine the fate of hearts subjected to I/R. The elucidation of the signalling pathways of ROS, NO and H_2_S is likely to reveal novel molecular targets for cardioprotection that could be modulated by pharmacological agents to prevent I/R injury.

## 1 INTRODUCTORY REMARKS

Chemically reactive species containing oxygen and/or nitrogen (ie ROS and RNS) are produced in virtually all cells during both physiological processes and pathological conditions. The contribution of reactive species to physiological signalling or pathological alterations depends on the frequency, intensity and duration of their availability. Indeed, a transient and mild increase in ROS/RNS levels is required for the intracellular transduction of several hormonal stimuli, whereas a prolonged and large increase is likely to cause profound derangements of cellular structures due to oxidative alterations of carbohydrates, lipids, proteins and nucleic acids. Changes in ROS/RNS levels are generally the result of an increased formation (or exposure to exogenous oxidants) along with a decrease in antioxidant defences. The term oxidative stress is commonly used to define a condition of imbalance between generation and removal of ROS or repair of resulting oxidative damage,^1^ along with less frequent terminology of nitrosative or nitro‐oxidative stress to describe RNS accumulation.

A solid and mechanistic characterization of ROS/RNS involvement in a given process is usually provided by the combination of the following approaches: (a) measurement of ROS/RNS levels; (b) assessment of oxidative changes of relevant targets; and (c) inhibition of the process of interest by antioxidant interventions (ie compounds or genetic manipulations). Not only are these approaches rarely used together, but also each of them has intrinsic limitations that hamper data interpretation, as well as the reliability of several studies in the field.^2^ For instance, besides methodological issues for ROS/RNS detection, due to their transient nature ROS/RNS levels might appear normal when oxidative damage is already produced, especially in intact organs or living animals (not to mention clinical studies).^3^ Regarding oxidative alterations of biomolecules, the causal relationships with a given phenomenon can hardly be defined in vivo, unless it is caused by a ROS/RNS source that can be specifically inhibited by pharmacological or genetic approaches. From a clinical point of view, it is worth pointing out that many biomarkers are available for detecting oxidative stress in plasma, yet their prognostic value is questionable.^4^

Despite these methodological and conceptual limitations, an increase in ROS and/or RNS has been linked to essentially any type of cardiac disease. This concept holds especially valid for oxidative stress supported by countless experimental data and many epidemiological studies. However, clinical trials, mostly carried out with non‐specific antioxidants, have so far failed to prove both a causal role for oxidative stress and the beneficial effects of its decrease.^5^

As far as the heart is concerned, considering the large fraction of the cardiomyocyte volume occupied by mitochondria that utilize more than 90% of oxygen reaching the cardiac muscle, it is hardly surprising that ROS generation occurs in mitochondria, thus making them an inevitable target of reactive species involved in cardiac pathophysiology. Mitochondria are also key targets of signalling pathways involved in cardioprotection.^6^, ^7^ In this respect, a crucial role is attributed to redox signalling generated by ROS, NO and hydrogen sulphide (H_2_S) that act mainly by means of covalent changes of target proteins and lipids.^8^

This review aims at providing a critical analysis of formation and role of reactive species in mitochondria, the role of NO and H_2_S with a special emphasis on mechanisms of injury and protection that determine the fate of hearts subjected to ischaemia and reperfusion (I/R).

## 2 MITOCHONDRIA AND ROS

### 2.1 Sources and targets

Mechanisms including specific enzymes responsible for mitochondrial ROS formation have been described by many excellent reviews.^6^, ^9^, ^10^, ^11^ Briefly, the mitochondrial formation of superoxide and hydrogen peroxide (H_2_O_2_) is catalysed by 16 (or more) different enzymes.^6^, ^10^ In most cases, ROS formation is a side, possibly undesired, reaction, especially at flavin or quinone sites of various enzymes or respiratory chain complexes.^12^ However, mitochondria contain also enzymes that generate H_2_O_2_ as an obligatory product of their catalytic activity. This is the case with p66^Shc^,^13^, ^14^ monoamine oxidases (MAOs)^15^ and possibly nicotinamide adenine dinucleotide phosphate oxidase 4 (NOX4),^16^ although its mitochondrial localization in cardiomyocytes is controversial.^17^ Quantitative comparisons among the various ROS sources can be carried out in isolated mitochondria.^12^, ^18^ However, the procedures to isolate mitochondria may result in several artefacts, for example in mitochondrial protein quantification.^19^ The contribution of the different enzymes to the overall mitochondrial ROS formation can hardly be quantified in intact cells and not at all in intact hearts or in vivo. This is because for most of ROS‐producing enzymes, loss‐of‐function studies carried out by means of inhibitors or genetic manipulations would inevitably compromise mitochondrial bioenergetics, eventually hampering the maintenance of cell viability. Actually, if ROS were produced just, or mostly, by respiratory chain complexes, as stated in countless articles, it would prove impossible to demonstrate that mitochondria produce ROS in vivo. This evidence has been obtained by pharmacological and genetic approaches targeting ROS sources, such as MAOs and p66^Shc^, the inhibition of which does not affect mitochondrial bioenergetics.^15^ Nevertheless, the question remains whether each pathway provides a fractional contribution to the overall ROS generation in mitochondria that would result from a sum of activities. The contribution of the various sources could vary in different pathological conditions. Alternatively, a cross‐talk among the various pathways exists, whereby the activation of some ROS sources modulates the activity of the other enzymes. This concept was supported by showing that combination of MAO inhibition, p66^Shc^ deletion and antioxidant treatment do not provide any additive effect on the decrease of both oxidative stress and I/R‐induced cardiac injury.^20^

The continuous formation of ROS is counterbalanced by the synergistic action of superoxide dismutases (SOD) and peroxidases. H_2_O_2_ generated by SOD, as well as by MAOs, p66^Shc^ and NOX4, is handled by catalase, that is specific for H_2_O_2_ outside of mitochondria, and several peroxidases localized in various cellular compartments including mitochondria. Peroxidases utilize the thiol‐containing compounds glutathione (GSH and GSSG in its reduced and oxidized form, respectively) and thioredoxin (Trx) for reducing H_2_O_2_ into water. Within mitochondria, peroxide reduction is catalysed mostly by glutathione peroxidases (Gpx1 and 4) and peroxiredoxin 3 (Prx3) that is maintained in its active reduced form by Trx.^21^, ^22^, ^23^

Peroxidase activities are balanced by the action of various reductases to readily regenerate the thiol groups in Trx and glutathione at the expense of NADPH(H^+^) oxidation. Therefore, the maintenance of an optimal NADPH(H^+^)/ NADP^+^ ratio is necessary to fuel thiol‐dependent peroxidases with reducing equivalents. Within mitochondria, NADP^+^ reduction into NADPH(H^+^) is operated mostly by malic enzyme, nicotinamide nucleotide transhydrogenase transferring electrons from NADH to NADP^+^ that depends on mitochondrial membrane potential (Δψ_m_) and isocitric dehydrogenase that is activated by a rise in intramitochondrial [Ca^2+^].^6^ Thus, oxidative metabolism and mitochondrial function are coupled to the mitochondrial antioxidant system by maintaining a high NADPH(H^+^)/ NADP^+^ ratio. Besides this short‐term control of antioxidant enzymes, long‐term adaptations to increased ROS levels are under the control of transcriptional factors, such as hypoxia‐inducible factors (HIFs) and nuclear factor erythroid 2‐related factor 2 (Nrf2).^23^, ^24^, ^25^, ^26^

Since mitochondria represent a primary source of ROS, they are inevitably a primary target of oxidative stress. Oxidative alterations have been described for respiratory chain complexes and several other proteins, lipid components, especially cardiolipin, and nucleic acids.^27^, ^28^ Notably, ROS synergizing with Ca^2+^ favour the opening of the permeability transition pore that plays a crucial role in I/R injury and represents a major target for cardioprotective intervention,^29^ as also covered by another article of this same issue. ROS target not only mitochondria, but also any cellular compartment, so that conditions of severe oxidative stress are hardly compatible with cell survival. However, a slight increase in ROS formation plays a significant role in many physiological processes trough the modulation of several transducing pathways.^30^, ^31^, ^32^ Although a thorough description of these processes is beyond the scope of this review, it is worth pointing out that the protective efficacy of conditioning protocols is largely contributed by ROS and is abolished by antioxidants.^33^, ^34^, ^35^ In a hormetic fashion, the low level ROS generation appears to maintain mitochondrial function ^36^ in a process that has recently been shown to contribute to cardioprotection induced by remote ischaemic pre‐conditioning.^37^ On the other hand, a large increase in ROS formation even at sublethal levels hampers various cellular functions. In both, cardiac and skeletal muscles, ROS‐induced alterations have been reported for contractile proteins,^38^, ^39^ as well as for channels and transporters involved in intracellular Ca^2+^ homeostasis.^40^ Therefore, ROS are causally involved in contractile impairments characterizing not only I/R injury, but also various cardiac diseases and muscular dystrophy.^38^, ^39^, ^41^ The direct involvement of mitochondria in contractile abnormalities induced by oxidative stress has recently been demonstrated by using a compound, mitoParaquat, that causes a primary increase in mitochondrial ROS formation.^42^

Not only ROS alters Ca^2+^ homeostasis, but also an increase in intracellular [Ca^2+^] is invariably associated with increased ROS levels, as shown for instance by increasing pacing frequency both in vitro and in vivo.^43^, ^44^, ^45^ Although several mechanisms have been proposed,^40^, ^46^, ^47^ how an increase in [Ca^2+^] is paralleled by ROS accumulation, the underlying processes remain to be elucidated conclusively.

In conclusion, ROS formation occurs at various mitochondrial sites and is counteracted by a complex scavenging system in both acute and long‐term responses. ROS produced within mitochondria are involved in physiological and pathological processes that modulate signalling pathways, mitochondrial (dys)function, contractile abnormalities and cell death.

### 2.2 Protective efficacy and limits of antioxidant interventions

The cardioprotective efficacy of interventions aimed at decreasing mitochondrial ROS accumulation supports the involvement of mitochondrially generated oxidative stress in many, if not all, cardiac diseases.^6^ However, on a more general standpoint, the experimental efficacy of antioxidant interventions has hardly been matched by positive results in clinical studies.^5^ This failure might be generated by the use of non‐specific antioxidants that do not prevent ROS formation and might also remove the fraction of ROS involved in endogenous cardioprotective mechanisms,^48^ such as ischaemic pre‐conditioning,^33^ or adaptive immune response via mitochondrial ROS‐triggered activation of the NLRP3 inflammasome.^49^ For instance, a role of adaptive or signalling ROS is exemplified by H_2_O_2_ which when added at very low concentrations decreased ischaemia‐reperfusion injury in an isolated heart.^50^, ^51^, ^52^

So far, no clinical study has been carried out to test interventions aimed at counteracting mitochondrial ROS formation in cardiac diseases. Nevertheless, a wide array of experimental data demonstrates that cardioprotection can be obtained by targeting either ROS formation or removal. Non‐specific interventions include antioxidant compounds that are targeted to mitochondria by means of conjugation to a lipophilic cation, such as triphenylphosphonium groups.^53^ Protection against reperfusion injury or heart failure has been obtained with MitoTEMPO^54^, ^55^ or MitoQ.^56^, ^57^, ^58^ Beneficial effects were also obtained with the small peptide SS‐31 that binds to cardiolipin preventing its oxidation.^9^, ^59^

The use of antioxidants does not facilitate the identification of specific sources of ROS involved in cardiac pathophysiology. As mentioned above, the inhibition of respiratory chain complexes and enzymes involved in substrate oxidation would profoundly hamper energy‐linked processes necessary for the maintenance of cell viability. Nevertheless, the specific inhibition of enzymes not involved in respiration and ATP synthesis has been reported to afford cardioprotective effects while unambiguously demonstrating that mitochondrial ROS formation increases during and contributes to cardiac injury. This is especially the case with p66^Shc^ and MAOs (reviewed in Ref. [13, 60]). While p66^Shc^ can only be inhibited by its genetic down‐regulation, MAOs are inhibited by reversible and irreversible inhibitors specific for the A or the B isoform. Perhaps more importantly, several MAO inhibitors are clinically available for the treatment of neurological disorders.^61^, ^62^ To the best of our knowledge, at present MAO inhibition is the only therapeutic approach aimed at mitochondrial ROS formation with compounds in current clinical use.^62^, ^63^

Besides inhibiting ROS sources, the study of ROS removing enzymes greatly contributed to the demonstration of the relevance of mitochondrial ROS formation in cardiac pathophysiology. In a loss‐of‐function approach, Mn‐SOD deletion was shown to cause substantial oxidative stress associated with a lethal cardiomyopathy.^64^ On the other hand, gain‐of‐function studies with catalase expression in mitochondria resulted in lifespan extension and protection against hypertensive cardiomyopathy.^65^, ^66^

In conclusion, a decrease in mitochondrial ROS formation is likely to inhibit pathological processes, yet it might also hamper signalling pathways involved in endogenous protection. Therefore, antioxidant interventions should be developed to inhibit specifically enzymes involved in pathological ROS formation rather than using non‐specific scavengers.

## 3 MITOCHONDRIA, NO AND CARDIOPROTECTION

### 3.1 Generation, timing and sources

The gaseous transmitter nitric oxide (NO)—although being a free radical with an unpaired electron—is considered an endogenous cardioprotective agent with multiple targets.^67^, ^68^, ^69^ Sources of NO in the body are the nitrate‐nitrite‐NO pathway that is considered as an exogenous source, since diet is important for the nitrate/nitrite supply,^70^ and the endogenous cellular source due to enzymatic production by the various nitric oxide synthases (NOS) using oxygen and L‐arginine as substrates.^71^ Besides conversion of nitrate to NO by bacteria in the oral cavity and gastrointestinal tract as well as reduction of nitrite by xanthine oxidase or reduced haemoglobin, inorganic nitrite reduction by mitochondrial cytochrome c oxidase was also reported.^72^ In partial contrast to the in vitro data, supplementation with nitrate in the in vivo situation seems to increase both mitochondrial biogenesis and efficiency of mitochondrial respiration (oxidative capacity).^70^

**FIGURE 1:**
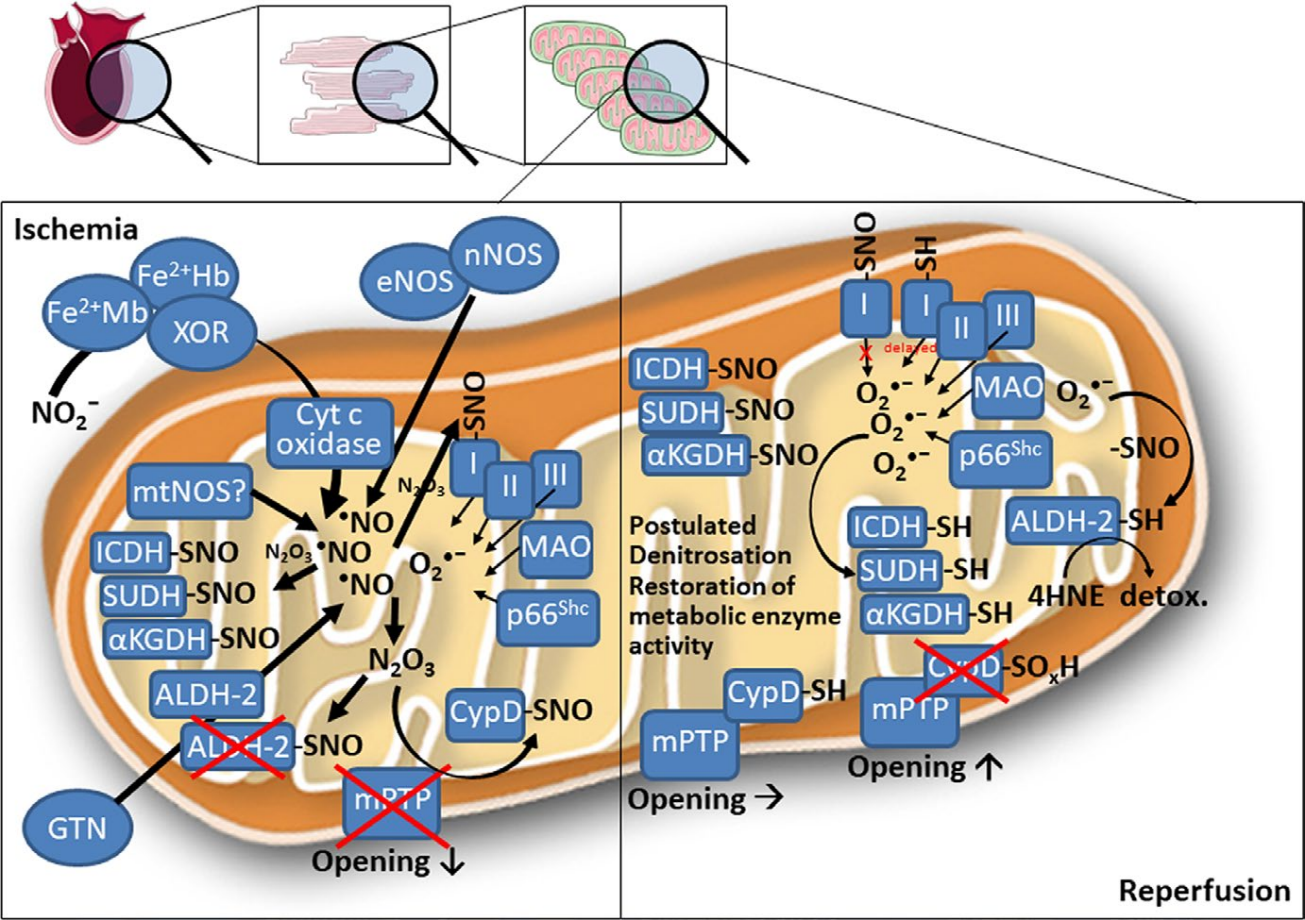
Proposed cardioprotective mechanisms of nitric oxide in cardiac mitochondria during ischaemia/reperfusion. During ischaemia, endogenous nitric oxide formation is potentiated from several sources. Mild nitrosative stress from inorganic nitrite conversion into nitric oxide, high activity of nitric oxide synthases (NOS) or pharmacological nitric oxide formation from nitroglycerin (GTN) combine with low superoxide levels from various mitochondrial sources to generate the potent nitrosating species N_2_O_3_. This leads to widespread nitros(yl)ation of mitochondrial enzymes involved in energy metabolism, as well as cyclophilin D (CypD). Nitros(yl)ated CypD cannot bind properly to the mitochondrial permeability transition pore (mPTP), thereby decreasing its open probability. Nitros(yl)ated enzymes involved in energy metabolism are inactive, yet nitros(yl)ation partially protects against irreversible oxidative damage. For instance, nitros(yl)ated aldehyde dehydrogenase 2 (ALDH‐2) limits GTN‐dependent NO formation in mitochondria thereby preventing severe nitrosative stress but also partially protects this important antioxidant enzyme against irreversible oxidative damage. Nitros(yl)ation of complex I limit infarct I/R injury by reducing/delaying superoxide formation at the onset of reperfusion. During reperfusion, superoxide formation from mentioned sources is increased and may lead to the postulated superoxide‐dependent denitrosation of enzymes involved in energy metabolism, thus restoring their activity for the required energy supply after an ischaemia/reperfusion episode. Superoxide‐dependent denitrosation of CypD restores its regulatory effect on mPTP favouring its opening. Superoxide‐dependent denitrosation of ALDH‐2 supports detoxification of cardiac damage by excessive formation of the detrimental 4‐hydroxynonenal (4HNE). αKGDH, α‐ketoglutarate dehydrogenase; ALDH‐2, mitochondrial aldehyde dehydrogenase; Fe^2+^Hb/Mb, ferrous haemoglobin/myoglobin; ICDH, isocitrate dehydrogenase; mtNOS, mitochondrial nitric oxide synthase; SUDH, succinate dehydrogenase; XOR, xanthine oxidoreductase. This scheme contains images from Servier Medical Art by Servier, licensed under a Creative Commons Attribution 3.0 Unported License

The two constitutive forms of NOS, endothelial NOS (eNOS, NOS3) and neuronal NOS (nNOS, NOS1) are present in the healthy heart, and the third form, the inducible NOS (iNOS, NOS2), is expressed as a response to inflammatory stimuli, such as prolonged myocardial ischaemia,^73^ and can also be involved in stress adaptation as illustrated in Figure 1, for example, by its appearance in the heart at the second window of protection after pre‐conditioning.^74^ The subcellular location of NOS in the cardiomyocyte relates to identified or proposed effects of NO. eNOS is mostly present in the caveolae—whereas the nNOS form is mostly seen close to or within the sarcoplasmic reticulum. A mitochondrial NOS (mitoNOS) has been indicated in several studies^75^, ^76^ and is most likely a nNOS subtype (NOS1). In humans, single nucleotide polymorphisms exist in the genes coding for the NOS enzymes (NOS1, NOS2 and NOS3). Interestingly, a nNOS (NOS1) polymorphism was associated with coronary heart disease suggesting that NOS is an important player in the pathology of cardiac I/R.^77^

Due to NOs‐free diffusion across biological membranes and its presence in blood and multiple targets, it has been difficult to pinpoint an exact cardioprotective mechanism. Moreover, the local concentrations of NO and superoxide determine whether NO is mainly converted to peroxynitrite and other RNS that can be detrimental^8^, ^78^, ^79^ or NO can exert its tissue protective effect.

In relation to mitochondria, cardioprotective mechanisms can be due to NO acting directly on the mitochondria or could be the result of indirect influence leading to protection of mitochondria. Interestingly, when exposing isolated mitochondria to NO, most studies report a decline in respiration as well as nitro‐oxidative damage of mitochondrial structures.^80^ Inhibition of complex IV (cytochrome *C* oxidase) and complex III (cytochrome *b* and *c*) has been described. A central concept is based on the reaction of NO with superoxide to produce peroxynitrite (ONOO^−^) promoting injury to the complexes and other molecules by nitration, thiol oxidation and redox changes in iron‐sulphur complexes,^80^ if not controlled by thiols and other scavenging molecules.^81^

Another general mechanism of mitochondria‐mediated cardioprotective effects of NO regarding the timing of NO formation and degradation is based on widespread *S*‐nitros(yl)ation of mitochondrial proteins involved in energy metabolism and apoptosis.^82^ Under hypoxic conditions, the lack of oxygen for mitochondrial respiration makes electrons from respiratory chain available for nitrite reduction into NO. The presence of superoxide triggers a complex multistep reaction whereby NO is converted into the potent nitrosating agent N_2_O_3_ leading to protein *S*‐nitros(yl)ation.^83^ During ischaemia, *S*‐nitros(yl)ated proteins are inactivated, but also protected against irreversible oxidative damage. Then during reperfusion, due to the increased superoxide formation *S*‐nitros(yl)ated proteins can be denitrosated recovering native structures and functions.

Mitochondrial matrix‐free calcium (Ca^2+^) plays a crucial role, stimulating enzymes in Krebs cycle and thereby supplying substrates for the respiratory chain. Interestingly, Ca^2+^ also activates NOS leading to formation of NO that due to its dampening effect on respiration plays a protective role in hypoxic conditions.^84^

In conclusion, the mechanism of cardioprotective effects of NO is based on a balance between ROS/RNS formation and degradation that may favour cardioprotective pathways, such as the NO‐cGMP (cyclic guanylate monophosphate)‐PKG (protein kinase G) axis and optimal *S*‐nitros(yl)ation of proteins, or stimulate cardiotoxic pathways at high ROS/RNS levels.

### 3.2 Therapeutic interventions

A variety of cardioprotective compounds protects mitochondria and increases NO by activating NOS. In addition to diffusion into the mitochondrial compartment, the activation of soluble guanylate cyclase by NO leading to cGMP and increase in PKG activity significantly contribute to mitochondrial protection. Activation of the cGMP/PKG pathway delays mitochondrial permeability transition pore (mPTP) opening preventing apoptotic cell death in cultured astrocytes.^85^ In addition, stimulation of the cGMP/PKG pathway reduces sarcoplasmic reticulum‐dependent calcium oscillations and thereby prevents hypercontraction and sarcolemmal rupture during the onset of reperfusion, also by beneficial regulation of mPTP opening.^86^, ^87^, ^88^ This interaction between the cGMP/PKG pathway and mPTP regulation could be exploited for pre‐ and post‐conditioning and improved cardiomyocyte survival during I/R.^89^ A major protective component of post‐conditioning may be the suppression of ROS formation at the onset of reperfusion, which will not only increase NO bioavailability and preserve functional cGMP/PKG signalling, ^90^ but also delay reperfusion‐dependent pH changes, ^91^ all of which is highly cardioprotective. Reports indicating the presence of the component of the cGMP‐PKG pathway in mitochondria add to the complexity, but also to the understanding of these processes.^92^ Likewise, BNP,^93^ bradykinin and insulin are examples of NO‐dependent activation of cytosolic PKG that then protects the mitochondria. Besides cGMP‐mediated mitochondrial cardioprotection, NO can also directly confer beneficial post‐translational changes as exemplified by inhibition of the mPTP via *S*‐nitros(yl)ation of cysteine 203 of CypD by *S*‐nitrosoglutathione.^94^ This process represents a highly attractive redox‐regulatory mechanism since hydrogen peroxide caused activation of CypD‐dependent mPTP opening via thiol oxidation of cysteine 203, which is obviously antagonized by nitric oxide‐dependent *S*‐nitros(yl)ation. This concept was recently exploited for cardioprotection by nitroglycerin administration to mice undergoing ligation‐induced myocardial infarction.^95^ Short‐term administration of nitroglycerin reduced the infarct size via increased CypD *S*‐nitros(yl)ation, whereas reduced infarct size was already present in CypD knockout mice. All protective effects of nitroglycerin were lost in mice rendered nitrate tolerant by chronic nitroglycerin administration and in eNOS knockout mice suggesting a vital cross‐talk between exogenous and endogenous NO formation,^96^ as well as NO reaction with superoxide to form peroxynitrite.

One of the key mechanisms of ischaemic pre‐ and post‐conditioning seems to be that mild oxidative stress activates antioxidant defence mechanisms (eg via Nrf2) that in the intermediate or long‐term time scale confer cardioprotection. A more direct antioxidant mechanism involved in NO‐mediated pre‐conditioning may be related to NO‐dependent *S*‐nitros(yl)ation of metabolic and survival key proteins in mitochondria, thereby protecting these proteins from irreversible thiol oxidation but also beneficially influencing mitochondrial oxygen consumption under hypoxic conditions (during the ischaemic phase).^82^ The *S*‐nitros(yl)ation of Cys39 of complex I was reported as a key mechanism of cardioprotection during I/R, which significantly limits myocardial infarction.^97^ During reperfusion, the generated superoxide will react with the *S*‐nitrosothiols and lead to reactivation of the enzymes via denitrosation. The optimal nitros(yl)ation conditions are reached when superoxide and nitric oxide are formed at a ratio of 1:3 leading to the formation of the potent nitrosating agent N_2_O_3_ and nitrite.^83^ This concept of ‘oxidative nitrosation’ is well accepted ^98^ and may explain the beneficial health effects of mild nitrosative/oxidative stress. This concept is supported by several earlier studies showing that mild oxidative/nitrosative stress triggers pre‐conditioning.^99^, ^100^, ^101^ This concept also goes very well with the mitochondrial bioactivation of nitroglycerin by mitochondrial aldehyde dehydrogenase (ALDH‐2),^102^, ^103^ allowing a direct mitochondrial action of nitroglycerin‐generated NO (eg on above described CypD/mPTP inhibition,^95^ as well as protective nitros(yl)ation of metabolic and survival key enzymes ^82^). These features would make nitroglycerin not only a fast‐acting antianginal drug and potent nitrovasodilator in stenotic areas of the heart,^104^ but also confer protection against I/R damage ^105^ by limiting the exemplified mitochondrial adverse effects, such as mPTP opening and oxidation of redox‐sensitive thiol groups leading to inactivation of metabolic key enzymes. Nitros(yl)ation of ALDH‐2 causes inactivation of the enzyme preventing excessive formation of NO nitroglycerin ^106^ and thereby severe cardiac nitrosative stress. In addition, *S*‐nitros(yl)ated ALDH‐2 would be protected from well‐established oxidative inactivation of the enzyme.^107^ Denitrosation of ALDH‐2 during reperfusion would allow detoxification of 4‐hydroxynonenal by ALDH‐2 conferring important cardioprotection during I/R.^108^, ^109^

In conclusion, the modulation of mitochondrial NO production and NO‐related cardioprotective mechanisms is a promising therapeutic option; however, further exploration of the complex interaction of NO, ROS and RNS is needed to design rational therapy. Further challenge of drug development is that major comorbidities of ischaemic heart disease, such as diabetes, hyperlipidemia and obesity, fundamentally change cardiac NO metabolism that may alter or even disrupt NO‐related cardioprotective pathways (for extensive reviews see ^8^, ^110^, ^111^, ^112^, ^113^, ^114^).

## 4 MITOCHONDRIA, H_2_S AND CARDIOPROTECTION

### 4.1 Generation, timing and sources

Hydrogen sulphide (H_2_S) is well‐recognized as a second messenger implicated in many physiological processes in mammals, including protection from oxidative stress.^115^, ^116^, ^117^, ^118^ The antioxidant effects of H_2_S have been conserved during evolution and described to operate in both prokaryotes and eukaryotes. H_2_S has been implicated in bacterial defence against ROS and in antibiotic‐induced oxidative damage.^119^ Partially through its antioxidant actions, H_2_S contributes to increased lifespan and anti‐ageing effects in yeast, *Caenorhabditis elegans* and *Drosophila melanogaster*.^120^, ^121^ In mammalian systems, reduced expression of endogenous H_2_S shifts cellular redox state towards a more oxidative state and administration of H_2_S donors lowers ROS levels in various cells and tissues.^115^ H_2_S can directly scavenge ROS (including hypochlorous acid, hydrogen peroxide, lipid hydroperoxides) and RNS, such as peroxynitrite.^117^, ^118^ However, H_2_S is considered a poor ROS/RNS scavenger.^122^ The relevance of the direct scavenging effects of H_2_S in biological systems has been questioned as H_2_S levels are low (nmol/L) compared to other cellular antioxidants that exist in the μmol/L to mmol/L range. On the other hand, H_2_S has been proven to prevent irreversible cysteine overoxidation preserving protein function.^123^ In addition, H_2_S has a variety of indirect antioxidant effects, many of which are mediated by activation of the master‐regulator of antioxidant responses Nrf2.^124^ In the heart, H_2_S increases GSH synthesis and up‐regulates the expression of thioredoxin.^115^, ^125^ Studies have demonstrated that H_2_S may act as an endogenous antioxidant mediator by inhibition of p66^Shc^‐mediated mitochondrial ROS production, rather than via the direct quenching function.^126^ However, the importance of p66shc for cardiac I/R injury and cardioprotective interventions has recently been questioned.^14^, ^127^

**FIGURE 2:**
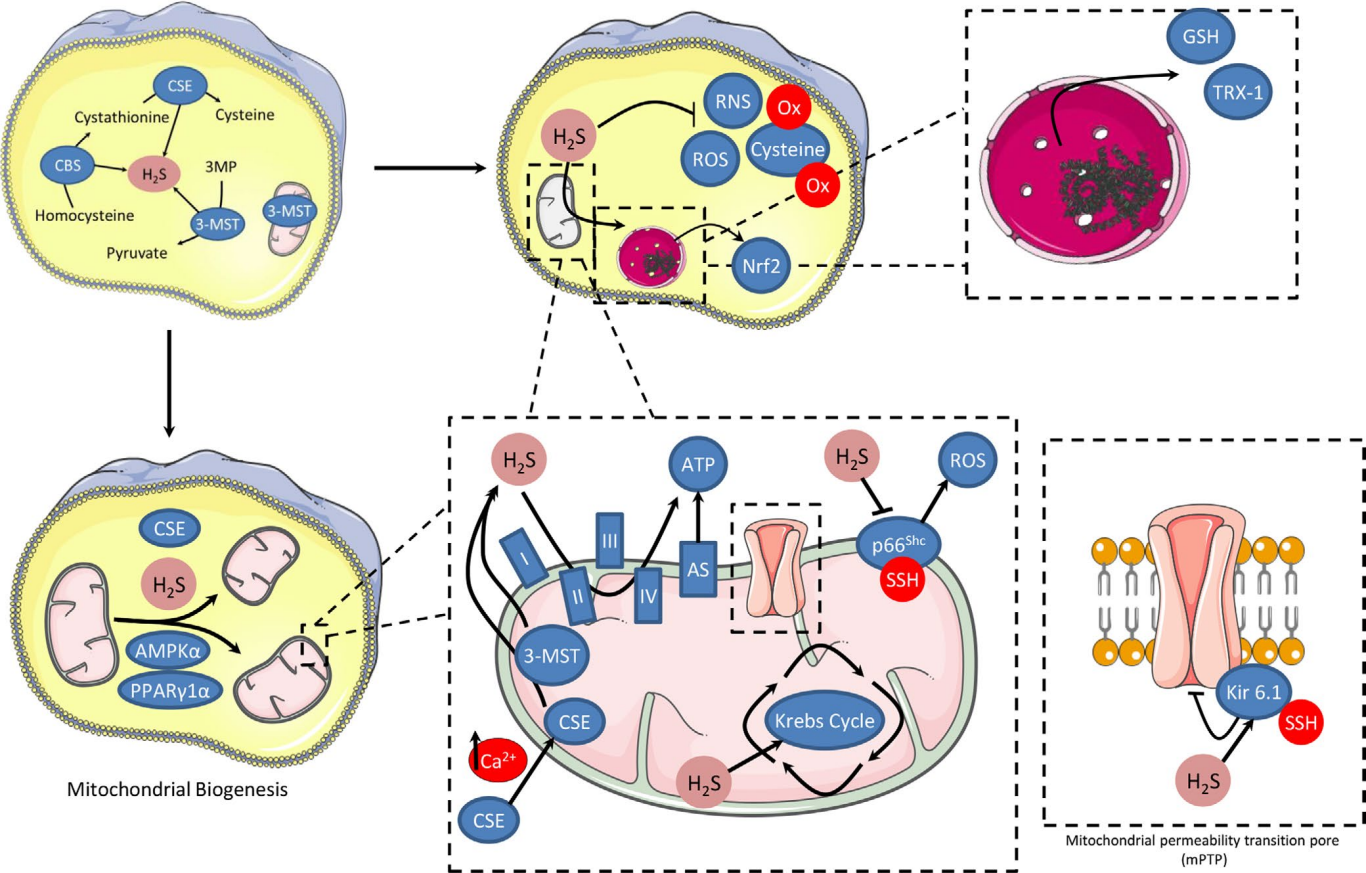
Proposed sources and targets for mitochondrial H_2_S generation involved in cardioprotection. H_2_S can be generated from 3‐mercaptopyruvate sulphurtransferase (3‐MST), that has been found in both cytosol and mitochondria and from the translocation of cystathionine γ‐lyase (CSE) from the cytosol to mitochondria after prolonged elevation of Ca^2+^ levels. H_2_S induces cardioprotection by preservation of mitochondrial function: H_2_S can inhibit ROS and RNS formation preventing irreversible cysteine overoxidation and preserving protein functions. H_2_S activates the master‐regulator of antioxidant responses Nrf2, increases glutathione (GSH) synthesis and up‐regulates the expression of thioredoxin. H_2_S may act as an endogenous antioxidant mediator by inhibition of p66^Shc^‐mediated mitochondrial ROS production. Another possible mechanism of action for H_2_S is based on its ability to modulate cellular respiration during reperfusion. Under physiological H_2_S concentrations, cytochrome c oxidase remains functional, whereas sulphide oxidation likely contributes to mitochondrial ATP production. Additionally, H_2_S regulates mitochondrial biogenesis by activation of AMP‐activated protein kinase and peroxisome proliferator‐activated receptor γ coactivator 1α. H_2_S modulates cellular signalling by sulfhydration, and among the proteins confirmed to undergo sulfhydration upon exposure to H_2_S, several are involved in cardioprotection including the pore forming subunit of ATP‐sensitive potassium channels (Kir 6.1)

One of the first protective effects of H_2_S in the cardiovascular system reported in the literature was its ability to limit I/R injury.^128^ Endogenously, H_2_S is mainly generated from three different enzymes: cystathionine β‐synthase (CBS), cystathionine γ‐lyase (CSE) and 3‐mercaptopyruvate sulphurtransferase (3‐MST), all of which are expressed in the heart (Figure 2).^124^ Under resting conditions, CSE and CBS are mainly present in the cytosol, while 3‐MST has been found in both the cytosol and the mitochondria.^129^ Mice overexpressing CSE were shown to have reduced infarct size compared to littermate controls.^128^ In contrast, obligatory CSE KO mice exhibited increased infarcts following I/R.^130^

Among other mechanisms, H_2_S‐induced cardioprotection involves preservation of mitochondrial function.^128^ A possible mechanism of action for H_2_S is based on its ability to modulate cellular respiration during reperfusion. Under physiological H_2_S concentrations, cytochrome c oxidase remains functional, whereas sulphide oxidation likely contributes to mitochondrial ATP production.^131^ It has been shown that H_2_S generated inside mitochondria by 3‐MST is sufficient to enhance mitochondrial electron transport and cellular bioenergetics.^132^ In vascular smooth muscle cells cultured under resting conditions, CSE is confined to the cytosol. However, prolonged elevation of calcium levels by the calcium ionophore A23187 leads to CSE translocation into mitochondria, increasing total H_2_S production in this organelle.^133^ If a similar phenomenon is also observed in cardiomyocytes, enhanced H_2_S output could help support ATP production under stress conditions.

The inhibition of mitochondrial respiration protects against I/R injury by limiting ROS generation and diminishing the degree of mitochondrial uncoupling leading to decreased infarct size and preserved contractile function.^134^, ^135^ When H_2_S was administered in vivo to mice at the time of reperfusion, the function of isolated cardiac mitochondria following 24 hours of reperfusion was better preserved, as noted by increased complex I and II efficiency. Electron microscopy revealed a striking reduction in mitochondrial swelling and increased matrix density in mice treated with a H_2_S releasing compound.^128^

In cardiomyocytes, interfibrillar (IFM) and subsarcolemmal (SSM) mitochondria are the two main types of mitochondria.^136^ Interestingly, in isolated rat hearts H_2_S preserves mitochondrial function and integrity especially in the IFM fraction.^131^, ^137^ Additionally, intramitochondrial H_2_S is essential for the citric acid cycle. Metabolite levels are altered during oxidative stress due to increased H_2_S degradation and reduced H_2_S production.^138^, ^139^

In addition to cellular bioenergetics, H_2_S was recently shown to regulate mitochondrial biogenesis. Cardiomyocytes of CSE‐deficient mice contained fewer mitochondria when compared to wild‐type hearts.^140^ In contrast, cardiomyocyte CSE overexpressing mice and mice receiving an H_2_S‐releasing prodrug exhibited enhanced mitochondrial content. H_2_S‐induced mitochondrial biogenesis involved activation of AMP‐activated protein kinase and peroxisome proliferator‐activated receptor γ coactivator 1α.

One of the main mechanisms through which H_2_S modulates cellular signalling is sulfhydration.^141^, ^142^ Sulfhydration is a post‐translational modification involving the addition of a thiol to a cysteine residue to form a persulphide (–SSH). Several proteins in the cardiovascular system become sulfhydrated, mediating the effects of H_2_S.^143^ Among proteins undergoing sulfhydration upon exposure to H_2_S, several ones are involved in cardioprotection including the pore forming subunit of ATP‐sensitive potassium channels (Kir 6.1), MEK1, p66^Shc^ and mitochondrial proteins.^143^, ^144^

In summary, H_2_S can be produced in cardiac mitochondria either directly by 3‐MST or through translocation of CSE from the cytosol to mitochondria under conditions of calcium overload and increased oxidative stress such as occurring in I/R. H_2_S can reduce I/R injury by preserving mitochondrial function and integrity, especially in the IFM fraction, potentially through post‐translational modifications of mitochondrial proteins.

### 4.2 Rationale for clinical use of H_2_S donors

One of the first protective effects of H_2_S in the cardiovascular system reported in the literature was its ability to limit I/R injury.^128^ As far as endogenous H_2_S is concerned, it has been demonstrated that mice overexpressing CSE were shown to have reduced infarct size compared to littermate controls.^128^ In contrast, obligatory CSE KO mice exhibited increased infarcts following I/R.^130^

Many groups have shown that H_2_S effectively ameliorates I/R injury by activating cardioprotective signalling pathways and by attenuating ROS and Ca^2+^ overload in mitochondria.^128^, ^130^, ^145^, ^146^, ^147^, ^148^ Depending on the nature of the H_2_S donor used, differences in the NO‐dependence of the cardioprotective effect have been noticed. The effects of fast releasing H_2_S donors, like NaHS and Na_2_S, are abolished in the presence of a NOS inhibitor, as well as in eNOS KO mice. In contrast, the action of donors that liberate H_2_S in a slow fashion (GYY4137 and thiovaline,^149^ or in a targeted manner (AP39, a mitochondrial donor), is NO‐independent.^146^, ^150^ Fast releasing donors reduce I/R injury by alleviating eNOS inhibition caused by the protein tyrosine kinase PYK2 phosphorylation of eNOS on Y656.^151^ NaHS pre‐conditioning significantly reduced myocardial infarct size and preserved the function of IFM; interestingly, the cardioprotective effects significantly declined in the presence of an inhibitor of endogenous H_2_S production (dl‐propargylglycine, PAG, CSE inhibitor).^152^ A mitochondrial H_2_S donor, AP39, reduced infarct size and significantly attenuated mitochondrial ROS generation, without affecting respiratory complexes I or II in SSM or IFM.^150^ In addition, AP39 increased the mitochondrial calcium retention capacity.^146^ Moreover, AP39 inhibited mPTP opening and reduced infarct size in mice lacking CypD, an activator of mPTP.^146^ Co‐incubation of mitochondria with AP39 and cyclosporine A, a pharmacological inhibitor of the CypD/mPTP interaction, induced an additive inhibitory effect on mPTP opening.^150^ Taken together, these results suggest that AP39 acts on mPTP at a site other than CypD binding site.

Among other mechanisms, H_2_S‐induced cardioprotection involves preservation of mitochondrial function.^128^ The inhibition of mitochondrial respiration protects against I/R injury by limiting ROS generation and diminishing the degree of mitochondrial uncoupling leading to decreased infarct size and preserved contractile function.^134^, ^135^ When H_2_S was administered in vivo to mice at the time of reperfusion, the function of isolated cardiac mitochondria following 24 hours of reperfusion was better preserved, as noted by increased complex I and II efficiency. Electron microscopy revealed a striking reduction in mitochondrial swelling and increased matrix density in mice treated with a H_2_S releasing compound.^128^

Diallyl trisulphide (DATS), a polysulphide found in garlic oil capable of releasing H_2_S, significantly reduced infarct size in mice subjected to I/R; DATS inhibited mitochondrial respiration in a concentration‐dependent manner and ameliorated mitochondrial coupling after reperfusion.^153^ At the same time, DATS activated eNOS and increased plasma nitrite and nitrate. Mitochondrial damage is a central feature of the intrinsic apoptotic pathway. Bax translocation to mitochondria contributes to the disruption of mitochondrial membrane potential and to the release of apoptotic proteins from the mitochondrial intermembrane space into the cytoplasm.^154^, ^155^ In mice subjected to I/R injury, Bax expression was reduced, while Bcl‐2 expression was increased in the hearts after treatment with NaHS.^156^ NaHS treatment also reduced the amount of activated caspase 3. In line with the above findings, fewer TUNEL‐positive cardiomyocytes were observed in the infarcted area in animals treated with NaHS.

In conclusion, the above‐mentioned findings provide a robust indication that direct delivery of H_2_S to mitochondria may represent a novel and effective intervention to mitigate the irreversible myocardial injury associated with I/R. This goal can be achieved either by treatment with mitochondria‐targeting H_2_S donors, such as AP39, or by conventional donors that increase cellular levels of H_2_S triggering cardioprotective pathways upstream of mitochondria.

## 5 UNSOLVED ISSUES AND FUTURE PERSPECTIVES

Despite the current knowledge of enzymes involved in mitochondrial ROS formation and removal, several questions remain to be solved for a complete understanding of the pathophysiological role of mitochondrial ROS in cardiomyocytes, as well as in other cell types.

Major issues appear to be as follows:

1. *ROS and other reactive species*. ROS also include peroxides that, especially in the case of lipids, might contribute to mitochondria and cell injury.^157^ In addition, when MAO activity is considered, along with H_2_O_2,_ very reactive aldehydes are generated.^158^ The cardioprotective effects granted by stimulating aldehyde dehydrogenase activity in mitochondria indicate the detrimental role of aldehydes.^159^ At present, it is not clear whether lipid peroxides and aldehydes are more relevant than superoxide and H_2_O_2_ in generating physiological and pathological effects (see also point 3).
2. *Relationships with mitochondrial bioenergetics*. A decreased activity of respiratory complexes with a concomitant increase in NADH(H^+^)/NAD^+^ ratio generally favours ROS formation due to superoxide generation at the level of complex I, II and III, including also reverse electron transport.^160^ Oxidative stress is also favoured by an increase in mitochondrial [Ca^2+^],^46^ perhaps in combination with Zn^2+^,^161^ opening of the mPTP^162^ or opening of mitochondrial K_ATP_ channels.^34^, ^163^ However, the molecular mechanisms by which these mitochondrial processes involved in I/R injury modulate oxidative stress are far from being elucidated. The question is especially relevant and complex in the case of mPTP opening, since it is both a consequence and a cause of ROS formation that appears to be involved either in the detrimental effects of post‐ischaemic reperfusion and in cardiac protection afforded by ischaemic pre‐conditioning.^164^
3. *ROS threshold*. In keeping with the previous point, it is generally accepted that a mild increase in ROS levels triggers protective mechanisms, while severe oxidative stress hampers cellular functions and viability. Indeed, it has recently been demonstrated that a primary formation of mitochondrial ROS induced by mitochondrially targeted paraquat (mitoPQ) causes cell death at high concentrations, yet a decrease of more than 10‐fold in mitoPQ levels protected against I/R injury.^42^ However, a quantitative method to determine the intracellular threshold separating beneficial from detrimental ROS is not yet available.
4. *Communication with the rest of the cell*. The fact that processes occurring in the cytosol affect mitochondrial function is documented by countless reports.^6^ This concept, including signalling pathways, ion homeostasis and proteostasis, holds also valid for oxidative stress. Indeed, ROS generated exogenously (ie toxicants or inflammatory processes) or within the rest of the cell (ie NOX activation) trigger mitochondrial responses by means of covalent changes in proteins, lipids and nucleic acids,^49^ as well as mitochondrial ROS formation. On the other hand, it is becoming clear that ROS generated within mitochondria are able to affect several cellular functions, including intracellular Ca^2+^ homeostasis and excitation‐contraction coupling,^9^, ^42^ as well as cytosolic and extra‐cellular ROS formation.^49^ Future studies should clarify the messages released to the rest of the cell downstream of an increase in mitochondrial ROS formation. Due to the abundance of SODs and peroxidases, the possibility that superoxide and especially H_2_O_2_ spread from mitochondria into the cytosol appears hardly tenable. On the other hand, the molecular mechanisms should be clarified by which an initial increase in cytosolic ROS levels is amplified by mitochondria.^49^, ^165^, ^166^
5. *Contributions from cells other than cardiomyocytes*. The abundance of mitochondria in cardiomyocytes has hindered the interest in mitochondrial formation of reactive species in other cell types of the heart. Although the available information is limited, mitochondria from non‐myocytes appear to play a significant role in cardiac pathophysiology. For instance, endothelial mitochondria generate ROS and RNS that are likely to be involved in both vascular and cardiomyocyte responses to physiological and pathological stimuli.^167^, ^168^ Although likely, reactive species cross‐talk between vascular cells and cardiomyocytes is far from being defined. The same applies to cells involved in inflammatory and immune responses that are present in any disease of the heart. An additional contribution as both source and target of reactive species is likely to be provided by fibroblasts, the number of which increases significantly in failing hearts. Interestingly, recent reports show that knockdown of the mitochondrial uncoupling protein 2 (UCP2) in fibroblasts resulted in a decreased ROS formation ^169^ that, however, was not observed in cardiomyocytes lacking UCP2.^170^ Future studies should investigate whether antioxidant interventions elicit similar or different effects in the various cell types of the heart under physiological and pathological conditions.

## CONFLICT OF INTEREST

PF is a founder and CEO of Pharmahungary Group, a group of R&D companies.
